# Rickettsiae in red fox (*Vulpes vulpes*), marbled polecat (*Vormela peregusna*) and their ticks in northwestern China

**DOI:** 10.1186/s13071-021-04718-1

**Published:** 2021-04-19

**Authors:** Gang Liu, Shanshan Zhao, Wenbo Tan, Sándor Hornok, Wumei Yuan, Ligu Mi, Suwen Wang, Zhiqiang Liu, Yanyan Zhang, Wurelihazi Hazihan, Xinli Gu, Yuanzhi Wang

**Affiliations:** 1 Department of Basic Medicine, School of Medicine, Shihezi University, Shihezi, 832003 Xinjiang, Uygur Autonomous Region China; 2 Department of Parasitology and Zoology, University of Veterinary Medicine, Budapest, Hungary; 3 Institute of Veterinary Medicine, Xinjiang Academy of Animal Science, Urumqi, Xinjiang, Uygur Autonomous Region China; 4 State Key Laboratory of Sheep Genetic Improvement and Healthy Production, Institute of Animal Husbandry and Veterinary, Xinjiang Academy of Agricultural and Reclamation Science, Shihezi, Xinjiang, Uygur Autonomous Region China; 5 Department of Veterinary Medicine, College of Animal & Science, Shihezi University, Shihezi, Xinjiang, Uygur Autonomous Region China

**Keywords:** *Rickettsia*, Red fox, Marbled polecat, Ticks, Northwestern China

## Abstract

**Background:**

Previously, twelve *Rickettsia* species were identified in ticks, fleas, sheep keds (*Melophagus ovinus*), bats (*Pipistrellus pipistrellus*) and a tick-bitten patient in the Xinjiang Uygur Autonomous Region (XUAR) in northwestern China. Here we aimed to molecularly detect rickettsial agents in red fox (*Vulpes vulpes*), marbled polecat (*Vormela peregusna*) and their ticks.

**Methods:**

During 2018–2019, 12 red foxes, one marbled polecat and their ticks were sampled in two counties and a city of the XUAR. The heart, liver, spleen, lung and kidney of these 13 carnivores were dissected, followed by DNA extraction. Hard ticks were identified both morphologically and molecularly. All samples were examined for the presence of rickettsiae by amplifying four genetic markers (*17-kDa, gltA, ompA, sca1*).

**Results:**

A total of 26 adult ticks and 28 nymphs (38 *Ixodes canisuga*, nine *Ixodes kaiseri*, six *Haemaphysalis erinacei* and one *Dermacentor marginatus*) were collected from red foxes, and four *Ha. erinacei* ticks were removed from the marbled polecat. Analysis of cytochrome *c* oxidase subunit I (*COI*) gene sequences indicated that 2–32 nucleotides differed between *I. canisuga*, *I. kaiseri* and *Ha. erinacei* from northwestern China and Europe. *Rickettsia raoultii* was detected in three red foxes, *Candidatus* Rickettsia barbariae in a red fox, *Rickettsia sibirica* in a red fox and a marbled polecat, and *R. raoultii* in two tick species (*I. canisuga* and *D*. *marginatus*).

**Conclusions:**

To the best of our knowledge, *I. canisuga* and *I. kaiseri* have not been previously reported from red foxes in China. The DNA of *R. sibirica* and *R. raoultii* was detected for the first time in the organs of red foxes, and *R. sibirica* in the organs of a marbled polecat. This is also the first molecular evidence for the presence of *R. raoultii* in *I. canisuga*. Our findings expand the range of tick-borne pathogens in wildlife species and associated ticks in China.
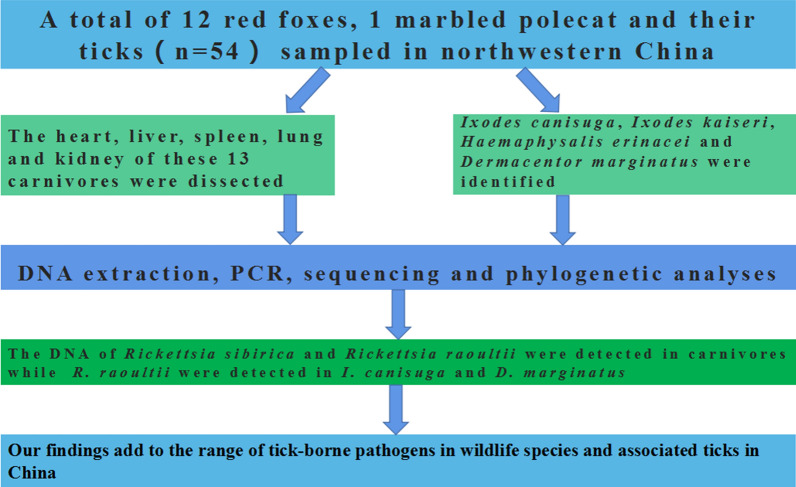

**Supplementary Information:**

The online version contains supplementary material available at 10.1186/s13071-021-04718-1.

**Background**

The red fox (*Vulpes vulpes*) is widely distributed throughout Europe, Asia, North Africa and North America [1]. Its habitats strongly overlap with those of other wildlife species, domestic animals and even humans [2]. Previously, red foxes were reported to harbor several vector-borne pathogens of public health concern, including tick-borne encephalitis virus [3], *Borrelia burgdorferi* [4], *Ehrlichia canis* [5], *Leishmania infantum* [6], *Hepatozoon canis* [7] and *Babesia vulpes* [8, 9]. A serological investigation of red foxes in Spain indicated that 50.3% had antigens of spotted fever group rickettsiae, including *Rickettsia massiliae* and *R. conorii* [10]. In addition, immunofluorescence assay showed that 1.9% of red foxes had antibodies to *R. typhi*, and 6.7% to *R*. *slovaca* in Spain [4].

The geographic range of the marbled polecat (*Vormela peregusna*) covers Central Asia, northwestern China and Europe [2]. In studies on its epidemiological role, seroconversion to plague F1 antigen was detected in a marbled polecat in the Xinjiang Uygur Autonomous Region (XUAR) [11] in northwestern China. *Borrelia burgdorferi *sensu lato and *Babesia* sp. were molecularly identified in a marbled polecat in Romania and China, respectively [12, 13]. Furthermore, *R*. *raoultii* and *Candidatus* Rickettsia barbariae were molecularly identified in marbled polecats in the XUAR [13].

In the temperate climate zone, hard ticks (Acari: Ixodidae) are regarded as the most important vectors of pathogens [14]. Among these, *Ixodes persulcatus*, *I*. *ricinus*, *I. hexagonus*, *I. kaiseri*, *I*. *canisuga*, *Dermacentor reticulatus*, *D. marginatus*, *Haemaphysalis punctata* and *Rhipicephalus sanguineus* have been reported from red foxes [10, 15–17]. In Spain, *R. massiliae*, *R. aeschlimannii* and *R. slovaca* were detected in red fox ticks [10]. In addition, *Ha. erinacei* from marbled polecats contained the DNA of *R. raoultii* in China [18].

In China, at least 19 spotted fever group (SFG) rickettsial species have been detected in ticks, including *R. heilongjiangensis*, *R. hulinii*, *R. mongolotimonae*, *R. sibirica* [19], *R. raoultii*, *R. slovaca* [20], *R. felis* [21], *R. aeschlimannii*, *R. massiliae* [22], *R. monacensis* [23], *R. japonica* [24], *Candidatus* R. barbariae, *R. conorii* [25], *R. parkeri*, *R. lusitaniae*, *R. rickettsii* [26], *Candidatus* R. jingxinensis, *Candidatus* R. tarasevichiae [27] and *Candidatus* R. leptotrombidium [28]. The aim of the present study was to investigate rickettsial agents in 12 red foxes, a marbled polecat and their ticks in China.

## Methods

### Sample collection and species identification

A total of 12 red foxes killed by illegal hunting or found as roadkill and one marbled polecat that died of natural causes were sampled in two counties and a city of the XUAR during 2018–2019 (data shown in Additional file 1). The red foxes and the marbled polecat were morphologically identified by an experienced zoologist. The heart, liver, spleen, lung and kidney of all 13 carcasses were removed. Simultaneously, the entire body surface of each individual was checked for ticks, all of which were removed. The ticks were morphologically identified to the species level according to the standard taxonomic keys as described previously [29]. This was also confirmed by molecular and phylogenetic analyses based on two mitochondrial markers, the 16S rRNA and the cytochrome *c* oxidase subunit I (*COI*) genes [17].

### Detection, sequencing and phylogenetic analysis of rickettsiae

Genomic DNA was extracted from the organs (heart, liver, spleen, lung and kidney) of wild carnivores as well as from their ticks using the TIANamp Genomic DNA Kit (TIANGEN, Beijing, China). To investigate the presence of rickettsiae in ticks, four genetic markers were targeted, including the 17 kDa antigen (*17-kDa*), citrate synthase (*gltA*), outer membrane protein A (*ompA*) and surface cell antigen 1 (*sca1*) genes. Two genes (*gltA* and *ompA*) were used to detect rickettsiae in the organs of wild carnivores [30]. The primers and polymerase chain reaction (PCR) cycling conditions used in this study are shown in Additional file 2. Each PCR assay included a negative control (distilled water instead of DNA template) and a positive control (containing sequence-verified DNA of *R. massiliae* from *Rh. turanicus* ticks collected in the XUAR) [22]. Purification and sequencing of the PCR products were performed as described previously [31, 32]. Sequences were manually edited, aligned and compared to reference GenBank sequences by the nucleotide BLASTn program (https://blast.ncbi.nlm.nih.gov). A phylogenetic tree was constructed using the maximum likelihood method in MEGA 7.0 software [18].

## Results

### Tick identification

A total of 26 adult ticks and 28 nymphs (i.e., 38 *I. canisuga*, 9 *I. kaiseri*, 6 *Ha. erinacei* and 1 *D. marginatus*) were collected from 12 red foxes, and four *Ha. erinacei* ticks were found on the marbled polecat. Morphological features are shown in Additional file 3: Fig. S1.

### Molecular and phylogenetic analyses

**Fig. 1:**
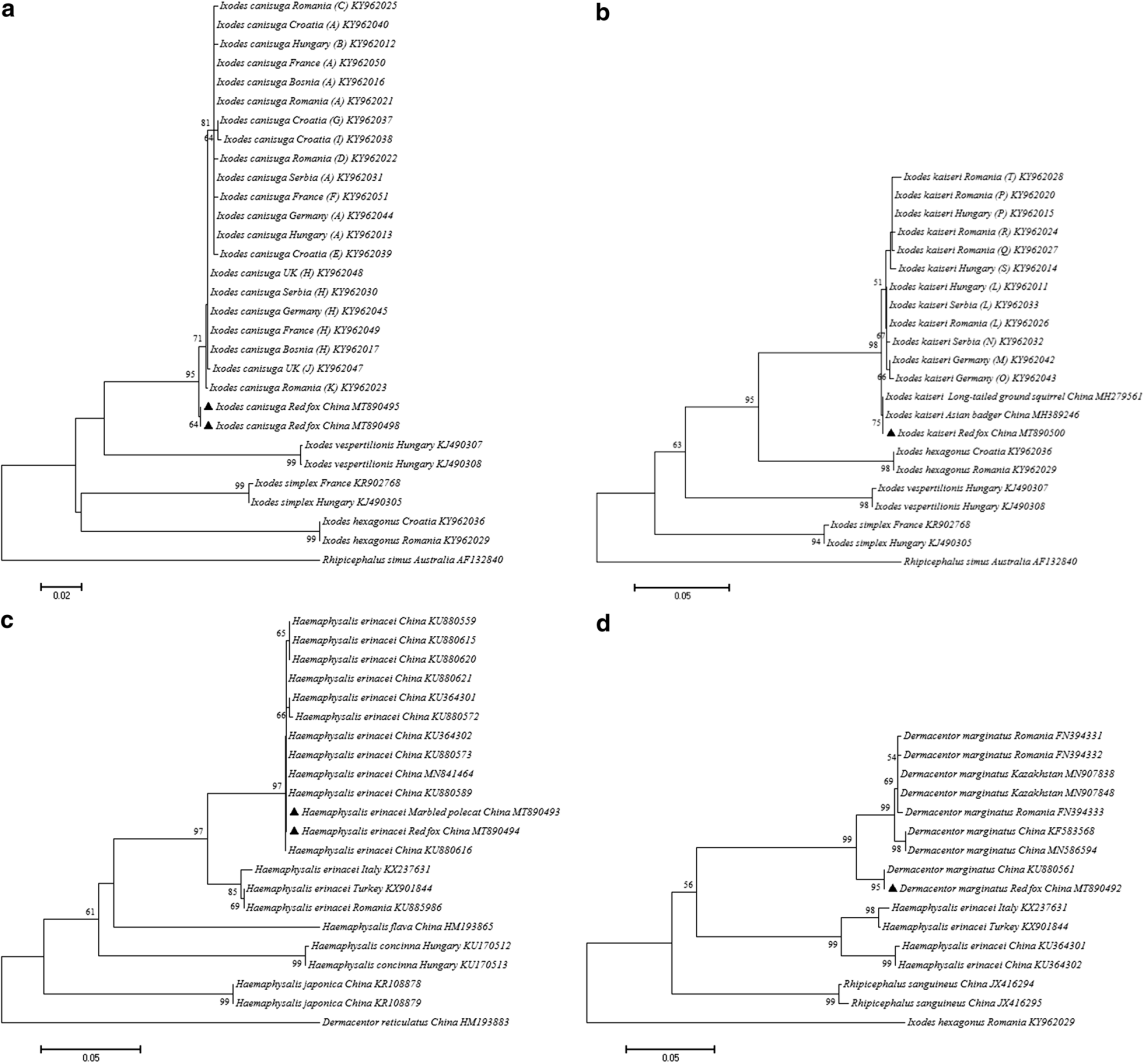
Phylogenetic tree based on *COI* sequences of ticks collected from 12 red foxes and a marbled polecat in northwestern China. The evolutionary history was inferred using the maximum likelihood method (bootstrap replicates: 1000) with MEGA 7.0. New sequences obtained in this study are indicated by black triangles. **a** *Ixodes canisuga*, **b** *Ixodes kaiseri*, **c** *Haemaphysalis erinacei* and **d** *Dermacentor marginatus*

Analysis of *COI* sequences revealed 2–32 nucleotide differences in the case of *I. canisuga* (3–6 bp), *I. kaiseri* (2–7 bp) and *Ha. erinacei* (30–32 bp) between Europe and China. Phylogenetic analysis showed that (i) *I. canisuga* in the XUAR was in a basal position to 11 European haplotypes (“A to K”) [17] (Fig. 1a); (ii) *I. kaiseri* from red foxes in the XUAR was also in basal position to nine European haplotypes (“L to T”), and had an identical sequence with conspecific ticks from long-tailed ground squirrels and Asian badgers [17, 30, 33] (Fig. 1b); (iii) *Ha. erinacei* from red foxes and marbled polecat had identical sequences, and formed a distinct clade from those reported in Turkey, Italy and Romania (Fig. 1c); and (iv) *D. marginatus* from red fox #2 had an identical sequence with the off-host tick collected formerly in Altaw City, XUAR.

**Fig. 2:**
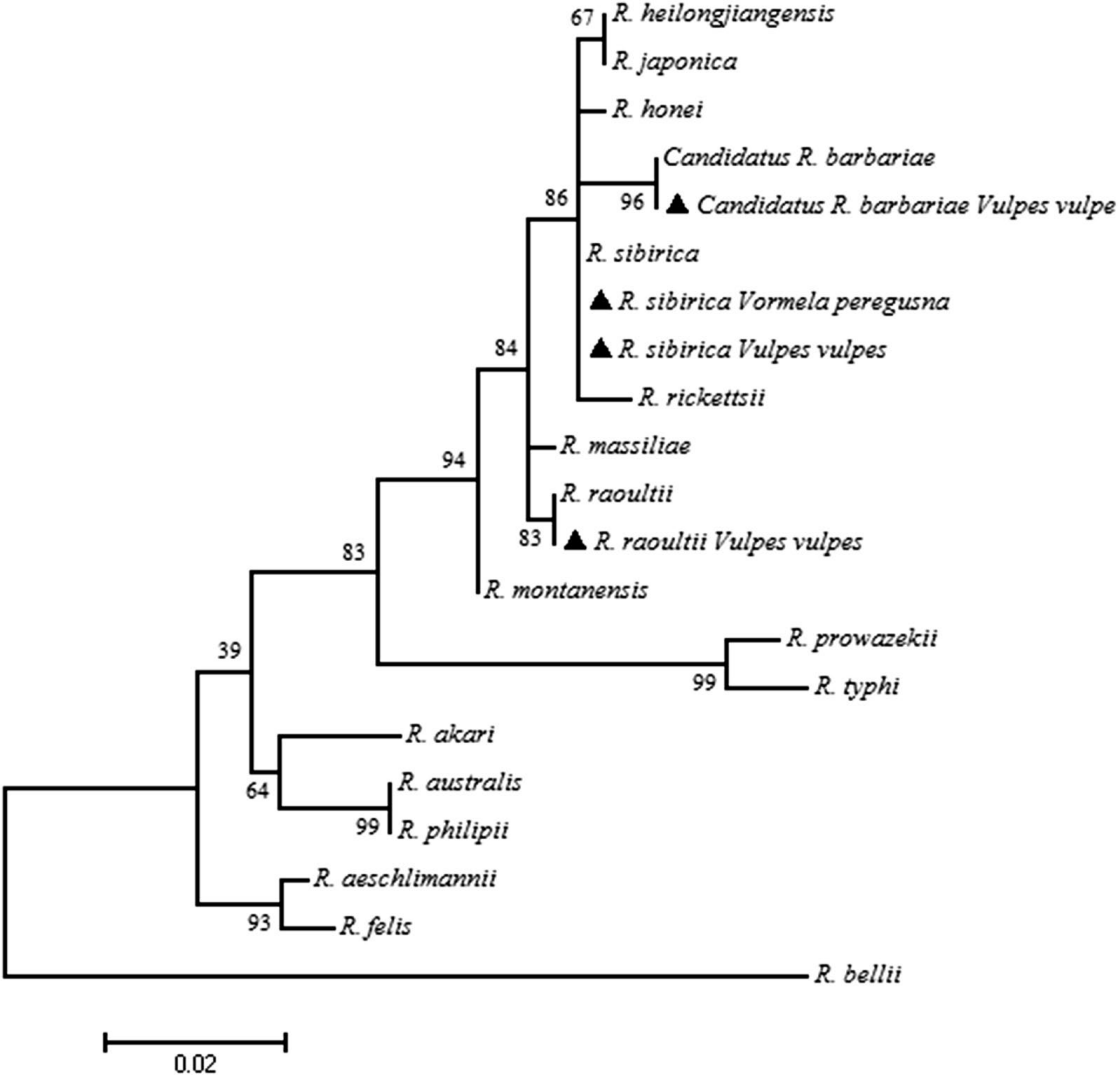
Phylogenetic tree of the *ompA*-*gltA* concatenated sequences of rickettsial agents in 12 red foxes and a marbled polecat

Red foxes #3, #5 and #11 were positive for *R. raoultii*, and red fox #8 was positive for *Candidatus* R. barbariae. At the same time, red fox #12 and the marbled polecat were positive for *R. sibirica*. In addition, *R. raoultii* was detected in *I. canisuga* from red fox #11 (Manas County) and *D*. *marginatus* from red fox #2 (Nilka County). Nucleotide sequences of rickettsial agents were deposited in the GenBank database (MT890502-MT890525). Phylogenetic analyses are shown in Fig. 2 and Additional file 4: Fig. S2.

## Discussion

Molecular studies on *I. canisuga* and *I. kaiseri* have been reported mostly from Europe, where these tick species typically infest Eurasian badgers (*Meles meles*), red foxes (*Vulpes vulpes*), steppe polecats (*Mustela eversmanni*), raccoon dogs (*Nyctereutes procyonoides*), hedgehogs (*Erinaceus europaeus*) and domestic dogs. Among these, dogs and red foxes can also be co-infested with *I. canisuga* and *I. kaiseri* [17, 34]. In this study, *I. canisuga* and *I. kaiseri* were found on red foxes, as also confirmed by 16S rRNA gene sequences (GenBank: MT889694-MT889698 and MT889701-MT889705). To the best of our knowledge, *I. canisuga* and *I. kaiseri* have been discovered here for the first time on red foxes in China. Phylogenetic analysis of the *COI* gene showed that (i) *I. canisuga* specimens collected from the same host species (red fox) were genetically different between China (MT890495-MT890498) and Germany (KY962044-KY962045), Croatia (KY962037-KY962040), Bosnia-Herzegovina (KY962016-KY962017), Serbia (KY962030-KY962031) and Romania (KY962025, KY962021-KY962023); (ii) *I. kaiseri* from red foxes and long-tailed ground squirrels in the XUAR had 100% identity, but clustered in a separate phylogenetic position compared to European ticks; and (iii) *Ha. erinacei* infesting the red fox and marbled polecat in the XUAR shared identical *COI* gene sequences (MT890493 and MT890494), but differed from those collected in Italy, Turkey and Romania (Fig. 1c). These findings support the contention that the genetic diversity of *I. kaiseri*, *I. canisuga* and *Ha. erinacei* might reflect geographic distribution rather than host associations, consistent with Klompen et al. [35].

In previous reports, *R. helvetica* was detected in the blood sample of a red fox in Switzerland [36]. In addition, *R. raoultii* and *Candidatus* R. barbariae were reported in a marbled polecat in Altaw City, the XUAR [13]. *R*. *raoultii* was also found in the tissues of bats (*Pipistrellus pipistrellus*) [26]. Here, *R. raoultii* was detected for the first time in the organs of red foxes, and *R. sibirica* in the lung and kidney of a marbled polecat and in the liver of a red fox.

Moreover, the DNA of *R. raoultii* was shown to be present in *I. canisuga.* Previously, *R. raoultii* has been detected in at least 15 different tick species, including *D. nuttalli*, *D. silvarum*, *D. marginatus*, *D. reticulatus*, *I. kaiseri*, *I. persulcatus*, *I. boliviensis*, *I. ricinus*, *Ha. concinna*, *Ha. japonica*, *Ha. erinacei*, *Rh. bursa*, *Rh. sanguineus*, *Hyalomma asiaticum* and *Amblyomma maculatum* [13, 18, 20, 27, 30, 37–44]. On the other hand, *R. sibirica* has been detected in at least 12 different tick species, including *Rh. turanicus*, *Rh. pusillus*, *Hy. asiaticum*, *Hy. truncatum*, *Hy. wellingtoni*, *Hy. yeni*, *D. marginatus*, *D. nuttalli*, *D. silvarum*, *D. sinicus*, *D. auratus* and *Ha. concinna* [22, 45–49]. Based on the above findings, the epidemiological role of red foxes, marbled polecats and their ticks in maintaining and transmitting rickettsiae can be suggested. However, further studies (including those on experimental transmission) are likely required to verify this hypothesis. In addition, the scope of this work should be extended to more wildlife species from China and Central Asia to better understand the epidemiological implications among rickettsial species and wildlife.

## Conclusions

To our knowledge, *I. canisuga* and *I. kaiseri* have not been previously identified from red foxes in China. The genetic diversity of *I. kaiseri*, *I. canisuga* and *Ha. erinacei* might be related to their geographic distribution rather than parasitized hosts. The results include the first detection of *R. raoultii* in *I. canisuga* and in red fox. We also provide the first molecular evidence of *R. sibirica* in red fox and marbled polecat. Our findings expand the range of tick-borne pathogens in wildlife species and associated ticks.

## Supplementary Information

**Additional file 1.** Sampling data of 12 red foxes (*Vulpes vulpes*), a marbled polecat (*Vormela peregusna*) and their ticks.

**Additional file 2.** Nested PCR protocol for the detection of rickettsiae in 13 wild carnivores as well as from their ticks, northern Xinjiang, China.

**Additional file 3.** The morphological characteristics of ticks from 12 red foxes and a marble polecat in northwestern China.

**Additional file 4.** Phylogenetic tree of the 17-kDa-ompA-gltA-sca1 concatenated sequences of *Rickettsia raoultii* from *Ixodes canisuga* and *Dermacentor marginatus* ticks.

## Data Availability

The sequences obtained and analyzed during the present study are deposited in the GenBank database under the accession numbers MT890502-MT890525 (Rickettsial), MT889693-MT889705 and MT890492-MT890501 (Tick 16S rRNA and *COI* gene).

## Abbreviations

COI: Cytochrome *c* oxidase subunit I
ompA: Outer membrane protein A
gltA: Citrate synthase
17-kDa: 17-KDa antigen
sca1: Cell surface antigen 1
XUAR: Xinjiang Uygur Autonomous Region
