# Supplementary material for: Rickettsiae in red fox (Vulpes vulpes), marbled polecat (Vormela peregusna) and their ticks in northwestern China
Source: Parasit Vectors. 2021 Apr 19;14:204. doi: 10.1186/s13071-021-04718-1 (PMC8054388; doi:10.1186/s13071-021-04718-1)
Supplement: Supplementary file 1 — Additional file 1. Sampling data of 12 red foxes (Vulpes vulpes), a marbled polecat (Vormela peregusna) and their ticks. [file 13071_2021_4718_MOESM1_ESM.docx]

**Additional file 1**. Sampling data of 12 red foxes (*Vulpes vulpes*), a marbled polecat (*Vormela peregusna*) and their ticks.

| **Serial number** | **Red fox** | | | | | | | | | | | | **Marbled polecat** |
| --- | --- | --- | --- | --- | --- | --- | --- | --- | --- | --- | --- | --- | --- |
|  | **#1** | **#2** | **#3** | **#4** | **#5** | **#6** | **#7** | **#8** | **#9** | **#10** | **#11** | **#12** |  |
| Age | adult | pup | pup | pup | pup | adult | adult | adult | adult | pup | pup | pup | adult |
| Habitat type | river valley | river valley | river valley | desert | river valley | river valley | desert | desert | desert | desert | desert | desert | desert |
| Location of origin | NC | NC | NC | AC | NC | NC | AC | AC | AC | MC | MC | MC | MC |
| *Haemaphysalis erinacei* |  |  |  |  |  |  |  |  |  |  | 2 | 4 | 4 |
| *Ixodes canisuga* |  |  |  | 4 |  |  |  |  |  | 10 | 11 | 13 |  |
| *Ixodes*  *kaiseri* | 1 | 4 | 3 |  | 1 |  |  |  |  |  |  |  |  |
| *Dermacentor marginatus* |  | 1 |  |  |  |  |  |  |  |  |  |  |  |

**Abbreviations:** NC Nilka County, AC Alataw City and MC Manas County
