# Supplementary material for: Rickettsiae in red fox (Vulpes vulpes), marbled polecat (Vormela peregusna) and their ticks in northwestern China
Source: Parasit Vectors. 2021 Apr 19;14:204. doi: 10.1186/s13071-021-04718-1 (PMC8054388; doi:10.1186/s13071-021-04718-1)
Supplement: Supplementary file 2 — Additional file 2. Nested PCR protocol for the detection of rickettsiae in 13 wild carnivores as well as from their ticks, northern Xinjiang, China. [file 13071_2021_4718_MOESM2_ESM.doc]

**Additional file 2**

**Nested PCR protocol for the detection of rickettsiae in 13 wild carnivores as well as from their ticks, northern Xinjiang, China**

The PCR equipment was a Mastercycler X50s, Eppendorf, Germany.

**1. Nested PCR amplification of the** *17kDa* **gene**

The nested PCR amplifications were performed in a 25-μL reaction volume. The reaction mixture contained 0.75 μmol/L of each primer, 250 μM of each dNTP, and 1.0 U of *Taq* polymerase (TaKaRa Taq Version 2.0, Takara, Dalian, China). Out:The cycling conditions consisted of an initial 5 min denaturation at 95°C, followed by 33 cycles at 95°C for 30 sec, 58°C for 60 sec, and 72°C for 1 min, with a final extension at 72°C for 8 min; In: The cycling conditions consisted of an initial 5 min denaturation at 95°C, followed by 33 cycles at 95°C for 30 sec, 61°C for 30 sec, and 72°C for 30 sec, with a final extension at 72°C for 8 min.

**2. Nested PCR amplification of the** *ompA* **gene**

The nested PCR amplifications were performed in a 25-μL reaction volume. The reaction mixture contained 0.75 μmol/L of each primer, 250 μM of each dNTP, and 1.0 U of Taq polymerase (TaKaRa Taq Version 2.0, Takara, Dalian, China). Out:The cycling conditions consisted of an initial 5 min denaturation at 95°C, followed by 35 cycles at 95°C for 30 sec, 50°C for 30 sec, and 72°C for 30 sec, with a final extension at 72°C for 8 min; In: The cycling conditions consisted of an initial 5 min denaturation at 95°C, followed by 35 cycles at 95°C for 30 sec, 59°C for 30 sec, and 72°C for 30 sec, with a final extension at 72°C for 8 min.

**3. Nested PCR amplification of the** *gltA* **gene**

The nested PCR amplifications were performed in a 25-μL reaction volume. The reaction mixture contained 0.75 μmol/L of each primer, 250 μM of each dNTP, and 1.0 U of Taq polymerase (TaKaRa Taq Version 2.0, Takara, Dalian, China). Out:The cycling conditions consisted of an initial 5 min denaturation at 95°C, followed by 37 cycles at 95°C for 40 sec, 50°C for 40 sec, and 72°C for 1 min, with a final extension at 72°C for 8 min; In: The cycling conditions consisted of an initial 5 min denaturation at 95°C, followed by 37 cycles at 95°C for 30 sec, 58°C for 30 sec, and 72°C for 1 min, with a final extension at 72°C for 5 min.

**4. Nested PCR amplification of the** *sca1* **gene**

The nested PCR amplifications were performed in a 25-μL reaction volume. The reaction mixture contained 0.75 μmol/L of each primer, 250 μM of each dNTP, and 1.0 U of Taq polymerase (TaKaRa Taq Version 2.0, Takara, Dalian, China). Out:The cycling conditions consisted of an initial 5 min denaturation at 95°C, followed by 35 cycles at 95°C for 30 sec, 50°C for 30 sec, and 72°C for 30 sec, with a final extension at 72°C for 8 min; In: The cycling conditions consisted of an initial 5 min denaturation at 95°C, followed by 35 cycles at 95°C for 30 sec, 55°C for 30 sec, and 72°C for 30 sec, with a final extension at 72°C for 5 min.

Characteristics of amplified fragments and corresponding primer sequences

| Species | [G](../../../../C:/Users/Administrator/AppData/Local/youdao/dict/Application/8.8.0.0/resultui/html/index.html#/javascript:;)ene | | [P](../../../../C:/Users/Administrator/AppData/Local/youdao/dict/Application/8.8.0.0/resultui/html/index.html#/javascript:;)rimer | [F](../../../../C:/Users/Administrator/AppData/Local/youdao/dict/Application/8.8.0.0/resultui/html/index.html#/javascript:;)ragment |  |
| --- | --- | --- | --- | --- | --- |
| *Rickettsia* | *17-kDa* | out | F-GCTTTACAAAATTCTAAAAACCATATA | 434 | [1] |
| R-TGTCTATCAATTCACAACTTGCCGTT |
| in | F-GCTCTTGCAACTTCTATGTT |
| R-CATTGTTCGTCAGGTTGGCG |
| *gltA* | out | F-ATGACCAATGAAAATAATAAT | 1178 | [1] |
| R-ATTGCAAAAAGTACAGTGAACA |
| in | F-GGAATCTTGCGGCATCGAGGATATG | 931 |
| R-CCATAGCTTTATAGATAATACCCG |
| *sca1* | out | F-GGTGATGAAGAAGAGTCTC | 657 | [1] |
| R-CTCTTTAAAATTATGTTCTAC |
| in | F-GAGGTTTGTGGATGCGTGGT | 553 |
| R-ACTGTGACTTTAGTACCGACA |
| *ompA* | out | F-ATGGCGAATATTTCTCCAAAA | 532 | [1] |
| R-AGTGCAGCATTCGCTCCCCCT |
| in | F-CTTAAAGCCGCTTTATTCACCACCTC | 433 |
| R-CCTGTATAATTATCGGCAGGAGC |

Reference

1. Zhao S, Yang MH, Jiang MM, Yan B, Zhao SS, Yuan WM, et al. *Rickettsia raoultii* and *Rickettsia sibirica* in ticks from the long-tailed ground squirrel near the China-Kazakhstan border. Exp Appl Acarol, 2019;77:425-433.
