# Supplementary material for: Rickettsiae in red fox (Vulpes vulpes), marbled polecat (Vormela peregusna) and their ticks in northwestern China
Source: Parasit Vectors. 2021 Apr 19;14:204. doi: 10.1186/s13071-021-04718-1 (PMC8054388; doi:10.1186/s13071-021-04718-1)
Supplement: Supplementary file 3 — Additional file 3. The morphological characteristics of ticks from 12 red foxes and a marble polecat in northwestern China. [file 13071_2021_4718_MOESM3_ESM.docx]

**Supplementary Figure 1.** The morphological characteristics of ticks from 12 red foxes and a marble polecat in northwestern China.


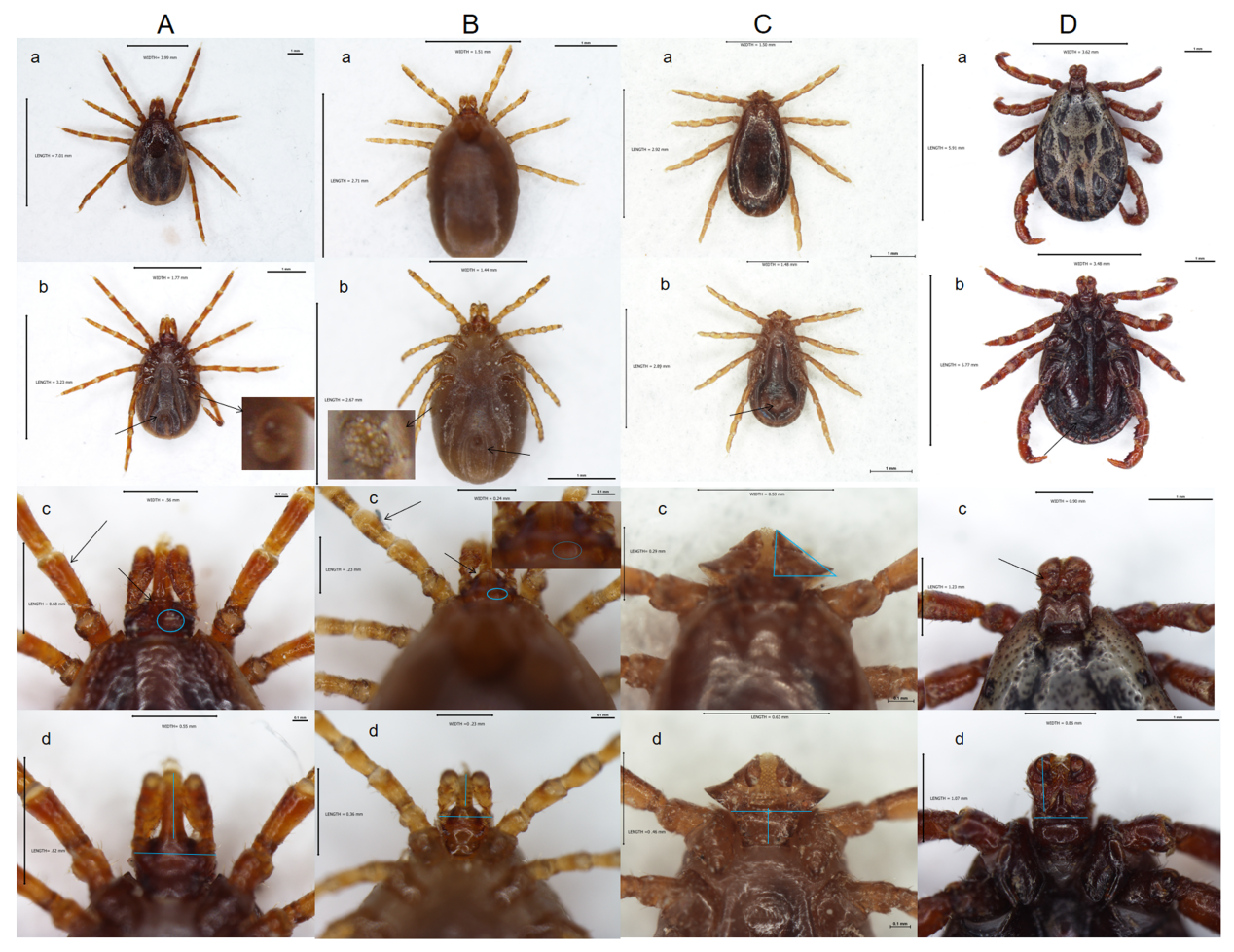


A. ***Ixodes canisuga***: legs with overall brown enameling; flat “plateau-like” anterior surface of basis capituli around the hypostome basis; porose areas subcircular, smaller; and hypostome of a similar length to that of the basis capituli.

**B. *Ixodes kaiseri***: legs with middle ring brown enameling; cone-like shape of the anterior surface of basis capituli; porose areas ovoid; and hypostome short, shorter length than width of the basis capituli.

**C. *Haemaphysalis erinacei***: palpal shape triangular; and the basis capituli short, much wider than long.

**D. *Dermacentor marginatus***: dorsal spur on palpal dorsal article II absent; and basis capituli wider or at least of the same width as the palpi.

**a**: Dorsal view. **b**: Ventral view. **c**: Basis capituli, dorsal view. **d**: Basis capituli, ventral view.
