# Supplementary material for: Rickettsiae in red fox (Vulpes vulpes), marbled polecat (Vormela peregusna) and their ticks in northwestern China
Source: Parasit Vectors. 2021 Apr 19;14:204. doi: 10.1186/s13071-021-04718-1 (PMC8054388; doi:10.1186/s13071-021-04718-1)
Supplement: Supplementary file 4 — Additional file 4. Phylogenetic tree of the 17-kDa-ompA-gltA-sca1 concatenated sequences of Rickettsia raoultii from Ixodes canisuga and Dermacentor marginatus ticks. [file 13071_2021_4718_MOESM4_ESM.docx]

**Supplementary Figure 2.** Phylogenetic tree of the *17-kDa*-*ompA*-*gltA*-*sca1* concatenated sequences of *Rickettsia raoultii* from *Ixodes canisuga* and *Dermacentor marginatus* ticks.


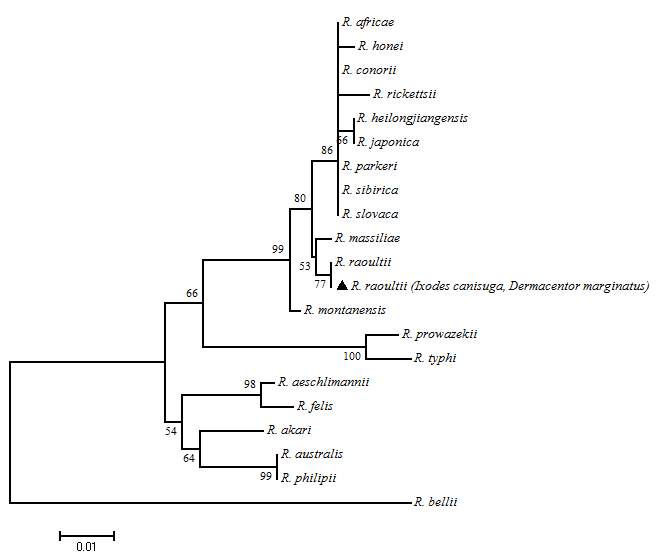


The tree was constructed with the Maximum Likelihood (bootstrap replicates: 1000) with MEGA 7.0. The concatenated sequence obtained in the present study is indicated by a black triangle.
